# Dataset for estimation of obesity levels based on eating habits and physical condition in individuals from Colombia, Peru and Mexico

**DOI:** 10.1016/j.dib.2019.104344

**Published:** 2019-08-02

**Authors:** Fabio Mendoza Palechor, Alexis de la Hoz Manotas

**Affiliations:** Universidad de la Costa, CUC, Colombia

**Keywords:** Obesity, Data mining, Weka, SMOTE

## Abstract

This paper presents data for the estimation of obesity levels in individuals from the countries of Mexico, Peru and Colombia, based on their eating habits and physical condition. The data contains 17 attributes and 2111 records, the records are labeled with the class variable NObesity (Obesity Level), that allows classification of the data using the values of Insufficient Weight, Normal Weight, Overweight Level I, Overweight Level II, Obesity Type I, Obesity Type II and Obesity Type III. 77% of the data was generated synthetically using the Weka tool and the SMOTE filter, 23% of the data was collected directly from users through a web platform. This data can be used to generate intelligent computational tools to identify the obesity level of an individual and to build recommender systems that monitor obesity levels. For discussion and more information of the dataset creation, please refer to the full-length article “Obesity Level Estimation Software based on Decision Trees” (De-La-Hoz-Correa et al., 2019).

**Table:** Specifications table

Subject area	*Biology*
More specific subject area	*Obesity, cardiovascular risk*
Type of data	*Text, table, figure*
How data was acquired	*Survey (see*Table 1*)*
Data format	*Raw and Analyzed*
Experimental factors	Data was retrieved from online survey and preprocessed including missing and atypical data deletion, and data normalization
Experimental features	*Labeling process was performed based on WHO and Mexican Normativity. Balancing class was performed using the SMOTE filter using the tool Weka. Features were chosen based on literacy analysis.*
Data source location	*Barranquilla – Colombia, Lima – Peru, City of Mexico - Mexico*
Data accessibility	*Data is within this article*
Related research article	*E. De-La-Hoz-Correa, F. Mendoza-Palechor, A. De-La-Hoz-Manotas, R. Morales-Ortega, B. Sánchez Hernández. Obesity Level Estimation Software based on Decision Trees, Journal of Computer Science, 67, 2019**[6]*

**Table:** 

**Value of the data** • This data presents information from different locations such as Mexico, Peru and Colombia, can be used to build estimation of the obesity levels based on the nutritional behavior of several regions. • The data can be used for estimation of the obesity level of individuals using seven categories, allowing a detailed analysis of the affectation level of an individual. • The structure and amount of data can be used for different tasks in data mining such as: classification, prediction, segmentation and association. • The data can be used to build software tools for estimation of obesity levels. • The data can validate the impact of several factors that propitiate the apparition of obesity problems.

## 1 Data

**Fig. 1:**
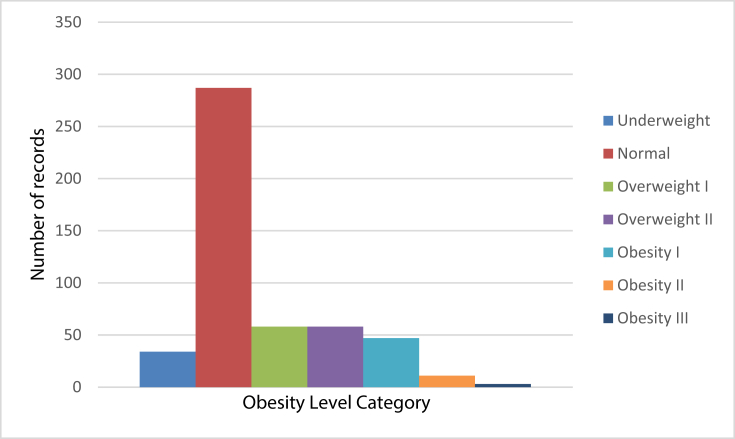
Unbalanced distribution of data regarding the obesity levels category.

**Fig. 2:**
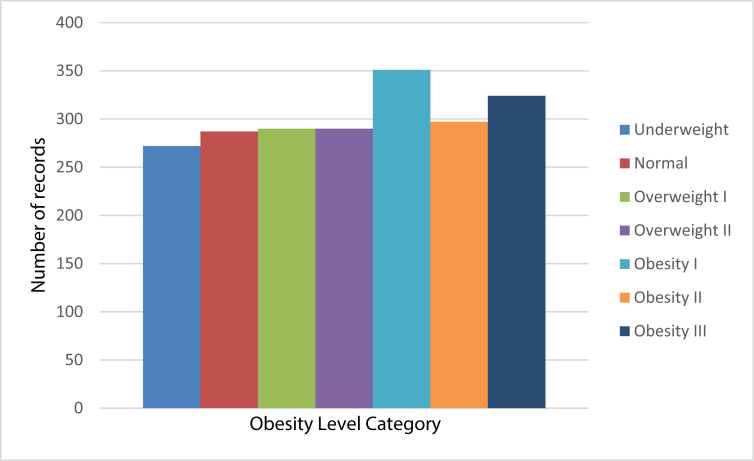
Balanced Distribution of data regarding the obesity levels category.

This paper contains data for the estimation of obesity levels in people from the countries of Mexico, Peru and Colombia, with ages between 14 and 61 and diverse eating habits and physical condition as mentioned by [1], data was collected using a web platform with a survey (see Table 1) where anonymous users answered each question, then the information was processed obtaining 17 attributes and 2111 records, after a balancing process described in Fig. 1, Fig. 2. The attributes related with eating habits are: Frequent consumption of high caloric food (FAVC), Frequency of consumption of vegetables (FCVC), Number of main meals (NCP), Consumption of food between meals (CAEC), Consumption of water daily (CH20), and Consumption of alcohol (CALC). The attributes related with the physical condition are: Calories consumption monitoring (SCC), Physical activity frequency (FAF), Time using technology devices (TUE), Transportation used (MTRANS), other variables obtained were: Gender, Age, Height and Weight. Finally, all data was labeled and the class variable NObesity was created with the values of: Insufficient Weight, Normal Weight, Overweight Level I, Overweight Level II, Obesity Type I, Obesity Type II and Obesity Type III, based on Equation (1) and information from WHO and Mexican Normativity. The data contains numerical data and continous data, so it can be used for analysis based on algorithms of classification, prediction, segmentation and association. Data is available in CSV format and ARFF format to be used with the Weka tool.

**Table 1:** Questions of the survey used for initial recollection of information.

Questions	Possible Answers
¿What is your gender?	• Female • Male
¿what is your age?	Numeric value
¿what is your height?	Numeric value in meters
¿what is your weight?	Numeric value in kilograms
¿Has a family member suffered or suffers from overweight?	• Yes • No
¿Do you eat high caloric food frequently?	• Yes • No
¿Do you usually eat vegetables in your meals?	• Never • Sometimes • Always
¿How many main meals do you have daily?	• Between 1 y 2 • Three • More than three
¿Do you eat any food between meals?	• No • Sometimes • Frequently • Always
¿Do you smoke?	• Yes • No
¿How much water do you drink daily?	• Less than a liter • Between 1 and 2 L • More than 2 L
¿Do you monitor the calories you eat daily?	• Yes • No
¿How often do you have physical activity?	• I do not have • 1 or 2 days • 2 or 4 days • 4 or 5 days
¿How much time do you use technological devices such as cell phone, videogames, television, computer and others?	• 0–2 hours • 3–5 hours • More than 5 hours
¿how often do you drink alcohol?	• I do not drink • Sometimes • Frequently • Always
¿Which transportation do you usually use?	• Automobile • Motorbike • Bike • Public Transportation • Walking

## 2 Experimental design, materials, and methods

The initial recollection of information was made through a web page using a survey where users had evaluated their eating habits and some aspects that helped to identify their physical condition. The survey was accesible online for 30 days. In Table 1, the questions of the survey are presented.

After all data was collected, then data was preprocessed, so it could be used for different techniques of data mining. The number of records was 485 records, and the data was labeled using equation (1).

(1) $$\;Mass\;body\;index=\frac{Weight}{height*height}$$

After all calculation was made to obtain the mass body index for each individual, the results were compared with the data provided by WHO and the Mexican Normativity [7].

• Underweight Less than 18.5
• Normal 18.5 to 24.9
• Overweight 25.0 to 29.9
• Obesity I 30.0 to 34.9
• Obesity II 35.0 to 39.9
• Obesity III Higher than 40

After the labeling process was finished, the categories of obesity levels were unbalanced (as shown in Fig. 1), and this presented a learning problem for the data mining methods, since it would learn to identify correctly the category with most records compared with the categories with less data. In [8], you can see a dataset is unbalanced if the classification categories are not represented equally.

After the balancing class problem was identified, synthetic data was generated, up to 77% of the data, using the tool Weka and the filter SMOTE proposed by [8]. The filter required to indicate the class for generation of synthetic data, the number of nearest neighbors used, the percentage that you need to increase the selected class and the random seed used for random sampling. Other aspects analyzed were the identification of atypical and missing data. Finally, after the filter was applied to each category, the final result were 2111 records. Next, in Fig. 2 you can see the final distribution of the data after the balancing process was completed.

It is important to notice that data must be preprocessed (delete missing data, atypical data, data normalization, etc.) before using SMOTE, since the neighbor selected to generate the synthetic data could contain noise or disturbances, and the data produced would have low quality. Nevertheless, using the filter SMOTE has a positive impact when data is unbalanced, since the balancing process decrease the probability of skewed learning on favor of a majority class.

Features included in the data were chose based in literacy analysis such as [1], [2], [3], [4], [5], [6], [8], and there is a noticeable relationship between weight and height given by Equation (1).

This data contributes to build tools using computational intelligence for detecting obesity levels based on eating habits and physical conditions, seeing that sometimes available data lack of the necessary number of records, they are not publicly accessible, and their structure makes difficult the application of several methods on the data.
